# Coitus-Free Sexual Transmission of Zika Virus in a Mouse Model

**DOI:** 10.1038/s41598-018-33528-2

**Published:** 2018-10-18

**Authors:** Chad S. Clancy, Arnaud J. Van Wettere, John D. Morrey, Justin G. Julander

**Affiliations:** 1 Utah Veterinary Diagnostic Laboratory, School of Veterinary Medicine, Department of Animal, Dairy, and Veterinary Sciences, Utah State University, Logan, Utah United States of America; 2 Institute for Antiviral Research, Department of Animal, Dairy, and Veterinary Sciences, Utah State University, Logan, Utah 84322-5600 United States of America

## Abstract

Zika virus (ZIKV) is an arboviral infection that may be sexually transmitted. The present study aims to determine if accessory sex glands are a potential source of infectious virus and important in sexual transmission. Male interferon type I receptor knockout (*Ifnar*^*−/−*^) mice were challenged subcutaneously with a Puerto Rican ZIKV isolate. Reproductive tissues were harvested seven days after viral challenge and artificial insemination fluid derived from epididymis or homogenized accessory sex glands (seminal plasma) was obtained. Naïve interferon type I and II receptor knockout (AG129) females were pre-treated with progesterone, and inoculated intravaginally with either epididymal flush or seminal plasma from ZIKV-infected males. ZIKV RNA was demonstrated in the artificial insemination fluid and ZIKV antigen was detected in epididymal epithelial cells but not within seminiferous tubules at the time of artificial insemination fluid collection. Peripheral viremia, demonstrated by ZIKV RNA in whole blood samples of females from each challenge group was observed. Infectious virus was present in both epididymal fluid and seminal plasma. These studies provide evidence of passage of virus from epididymal flush and seminal plasma to naïve females via artificial insemination and provides a model for the study of sexual transmission of ZIKV.

## Introduction

Zika virus (ZIKV) is an arbovirus that has recently emerged in the Western hemisphere. ZIKV is primarily transmitted by infected mosquitoes, however sexual transmission has also been documented^1–5^. ZIKV RNA can be detected in the semen of infected men up to 6 months following initial clinical signs^6,7^. Viable ZIKV has been isolated from spermatozoa^8^ and ZIKV virions from the ejaculate of symptomatic men are capable of infecting Vero cells during acute disease^9^. ZIKV RNA has also been detected by PCR from the ejaculate of vasectomized men^4^. Sexual transmission of ZIKV should be considered a possible source of further spread of virus in regions where primary mosquito vectors are not endemic.

The ejaculate is composed of secretions and contents of both the epididymis and accessory sex glands. Seminal plasma is the portion of ejaculate produced by the accessory sex glands, chiefly the seminal vesicle and the prostate. Seminal plasma is known to modulate the immune response. Seminal plasma in mice has been shown to suppress the humoral immune response by decreasing mitogen induced lymphocyte transformation^10^. Regulation of the female immune response via seminal plasma is due to, in part, cytokines TGF-β and prostaglandins^11,12^. A component of the seminal plasma produced by accessory sex gland epithelial cells is prostasomes, extracellular vesicles released primarily from prostatic epithelial cells but also from seminal vesicular epithelial cells^13^. Prostasomes have been shown to function in immunoregulation by inhibiting lymphocyte function, decreasing NK cell activity^13^ and preventing endocytosis by granulocytes and macrophages^14,15^. Prostatsomes have been hypothesized as a potential secretion pathway for enveloped viruses^16^. ZIKV RNA is detectable in homogenized accessory sex glands, primarily the seminal vesicle and the prostate gland^17^. A similar extracellular vesicle pathway exists in the epididymis, the epididymosome^13^. Epididymosome secretion is believed to occur in apocrine fashion^18^ similar to accessory sex glands. Prostasomes and epididymosomes may be a pathway for viral excretion from accessory sex gland epithelial cells.

Both male and female reproductive environments have implications for ZIKV infection. A 100-fold increase in progesterone sensitivity is observed in lymphocytes from pregnant female mice compared to non-pregnant females, with enhanced expression of nuclear progesterone receptors in peripheral blood lymphocytes^19^. Down-regulation of T-lymphocytes appears to be the primary route of systemic progesterone mediated immunosuppression^19^. Exogenous progesterone administration associated with increased circulating progesterone levels has previously been shown to enhance intravaginal infection of simian immunodeficiency virus^20^. Prolonged progesterone administration locally down-regulates mucosal immunity in response to herpes simplex virus type 2^21^. Progesterone has previously been shown to convey an immunosuppressive effect on mouse dendritic cells^22^, a cell type that has been shown to be permissive to ZIKV infection^23^. Supra-physiologic doses of exogenous progesterone enhances systemic ZIKV infection following intra-vaginal inoculation^24^.

In pregnant women, infection with ZIKV, particularly during early gestation^25^, is correlated with an increased risk of birth defects such as microcephaly^26^. Reports show that 5.8–8% of women infected with ZIKV during pregnancy had a child with a birth defect^25,27^. Development of an animal model to evaluate the potential for sexual transmission from infected males is important. Here, we show evidence of sexual transmission of ZIKV from seminal plasma and epididymal flush from ZIKV-infected, *Ifnar*^*−/−*^ (interferon type I receptor knockout) male mice to progesterone-treated, AG129 (interferon type I and II receptor knockout) female mice via artificial insemination utilizing a contemporary ZIKV isolate. We also show that supra-physiologic doses of progesterone mimicking lymphocyte sensitivity during pregnancy and not the cycle of estrous is important in predisposing females to intravaginal ZIKV infection.

## Materials and Methods

### Ethics Statement

Studies were conducted under approval of the Utah State University Animal Care and Use Committee protocol number 2598. Study approval and animal care was conducted in accordance with The Guide for Care and Use of Laboratory Animals^28^ and U.S. Government Principles for the Utilization and Care of Vertebrate Animals Used in Testing, Research and Training.

### Virus

A Puerto Rican isolate of ZIKV (PRVABC-59, BEI Resources, Manassas, VA, USA) was passaged two times in Vero 76 cells. Infected cells were frozen once, thawed, centrifuged to remove cell debris, aliquoted and stored at −80 °C. The virus stock had a titer of 10^7.0^ 50% cell culture infectious doses (CCID_50_/mL).

### Mouse studies

#### Male Infection and Sample Collection

Six to seven-week-old male *Ifnar*^*−/−*^ mice were inoculated subcutaneously with either sterile minimal essential media (MEM) solution (sham-infected; n = 6) or 10^2^ CCID_50_ of PRVABC59 ZIKV (n = 8) diluted in MEM. Samples were collected 7 days post infection (DPI). The left epididymis and vas deferens were isolated and placed in 500 µL of Tyrode’s media buffered to pH 7.6. The tail of the epididymis and vas deferens were opened with 27 gauge needles. The epididymal flush was incubated for 45 min at 37 °C with 5% CO_2_ enrichment. The left seminal vesicle and associated prostate were isolated, placed in 250 µL of Tyrode’s media, homogenized and centrifuged for 15 min at 4000 rpm. The supernatant was pulled off and this constituted the seminal plasma. Seminal plasma was coincubated with the epididymal flush for 5 min prior to female inoculation and this combination constituted the insemination fluid.

The remaining right testicle, epididymis, prostate, seminal vesicle and mouse carcass were placed in 10% neutral buffered formalin and fixed for 48 hours at room temperature.

#### Female Hormonal Treatment and Insemination

Female 8–10-week-old, AG129 mice were pre-treated with 2 mg of progesterone or placebo (sham-infected n = 10, progesterone-treated n = 60) as previously described^24^. Three days following progesterone administration, vaginal cytologies were prepared by gently douching the vaginal vault with 5 µL of sterile saline, and spreading fluid on a slide. Slides were allowed to air dry for 24 hours at room temperature, then stained routinely with Wright-Giemsa stain. The stage of estrus was determined as previously described^29^.

Six insemination treatments were created (Table 1). Treatment 1 comprised of material collected during an epididymal flush (EF) and the corresponding seminal plasma (SP) collected from a ZIKV-infected male. Treatment 2 was comprised of EF from a ZIKV-infected male (EF+) and SP from a sham-infected male (SP−). Treatment 3 included EF from a sham-infected male (EF-) and SP from a ZIKV-infected male (SP+). Treatment 4 was composed of EF- and SP- spiked with 10^4^ CCID_50_ of ZIKV. Females in treatment group 5 were challenged intravaginally with 5 × 10^3^ CCID_50_ of ZIKV diluted in MEM without any male reproductive components. Treatment 6 was comprised of EF- and SP-.

**Table 1:** Insemination Treatment Groups.

Treatment	Epididymal Flush (500 µL)	Seminal Plasma (250 µL)
1	Infected Male	Infected Male
2	Infected Male	Sham-infected Male
3	Sham-infected Male	Infected Male
4	Sham-infected Male/Spike	Sham-infected Male/Spike
5	10^4^ CCID_50_/100 μL ZIKV PRVABC59 in MEM	
6	Sham-infected Male	Sham-infected Male

Components of each treatment group. Each epididymal flush totaled 500 µl of fluid and seminal plasma totaled 250 µl of fluid. The epididymal flush and seminal plasma fluid were combined and coincubated creating the artificial insemination inoculum.; CCID_50_ = 50% cell culture infectious doses; MEM = Minimal Essential Media.

Three replicate groups of five progesterone-treated, AG129 females were intravaginally instilled with treatments 1, 2 and 3. One progesterone treated and one non-progesterone treated group (n = 5/group) of females was assigned to treatments 4 and 5. One progesterone treated group of females (n = 5) was assigned to treatment 6. Intravaginal instillation of 50 µL of insemination fluid was performed with an 18 gauge oral gavage needle. Weights and mortality of the females were recorded daily for 21 days thereafter.

#### Subcutaneous Infection

The intravaginal inoculation groups were replicated (n = 3 females/group) with a subcutaneous inoculation in the right hind leg of each mouse. The intravaginal inoculation fluid was diluted 1:10 in of sterile MEM. A 100 µL artificial insemination solution was deposited subcutaneously in the right hind leg of each mouse.

### Quantitative Reverse Transcriptase PCR

A 150 µL insemination fluid aliquot was frozen at −80 °C for RNA isolation and quantitative reverse transcriptase PCR (qRT-PCR). At 7 DPI, 200 µL of whole blood in EDTA was collected via cheek bleed from female AG129 mice in both intra-vaginal and subcutaneous inoculation groups. RNA was extracted using the Qiagen QIAamp cador Pathogen Mini Kit (Qiagen, Germantown, MD, USA) according to manufacture instructions and eluted with 100 µL of elution buffer.

Quantitative RT-PCR was performed as previously described^17^ using a Mic-2 qPCR thermocycler (Bio Molecular Systems, Coomera, QLD, Australia). Standard curves of ZIKV RNA and *GAPDH* RNA were generated with serial dilutions of synthetic RNA (GeneScript, Piscataway, NJ, USA) of the target sequence (accession HQ234499.1). The relative number of ZIKV RNA copies was determined by extrapolation from the standard curve, and normalized to *GAPDH* RNA for each sample.

### Histopathology

The severity of inflammation was analyzed in the male reproductive tract of sham-infected and ZIKV-infected males. Male reproductive tissues were processed routinely, embedded in paraffin wax and 3–5 µm thick serial sections were prepared. The first slide was stained with hematoxylin and eosin and the subsequent section was used for immunohistochemistry. Two veterinary anatomic pathologists (CSC and AJVW) independently and blindly scored the histologic lesion severity as previously described^17^.

### Immunohistochemistry

The male reproductive tract was analyzed for presence of ZIKV antigen using immunohistochemical staining. Tissue sections were deparaffinized and rehydrated. Epitope retrieval was achieved by placing slides in decloaking solution (Dako, Agilent Pathology Solutions, Santa Clara, CA), within a decloaking chamber (Biocare Medical, Pacheco, CA, USA) at 125 °C and 20 PSI for four minutes. The slides were cooled to room temperature then exposed to 0.5% triton for five minutes and washed three times in phosphate buffered saline (PBS) for 5 minutes each. Tissue sections were blocked for 10 minutes at room temperature with BLOXALL (Vector Laboratories, Burligame, CA) followed by incubation a 20 minute incubation with normal blocking serum (Vector Laboratories). Sections were then incubated with a rabbit polyclonal anti-Zika antibody diluted 1:500 in normal serum for 12 hours. Tissues were washed three times in PBS and incubated for 30 minutes in a goat anti-rabbit biotinylated secondary antibody solution (Vector Laboratories). Visualisation was achieved by incubating the slides with VECTASTAIN ABC-AP reagent (Vector Laboratories) for 30 minutes followed by a 5 minute wash in PBS and addition of alkaline phosphate substrate solution (Vector Laboratories) for 10–15 minutes with a final wash in tap water. Slides were counterstained with hematoxylin for 30 seconds. An aqueous mounting media was applied prior to sealing a coverslip.

### Statistical Analysis

Statistical analyses were performed in Microsoft Excel (Microsoft, Redmond, WA). A two-way ANOVA was conducted to determine statistically significant difference between the viral load in male-derived treatment (treatments 1, 2 and 3). A p value of ≤0.05 was used to determine significance. Statistical significance between individual groups was determined by a student’s T-test.

## Results

### Male Histopathology and Immunohistochemistry

**Figure 1:**
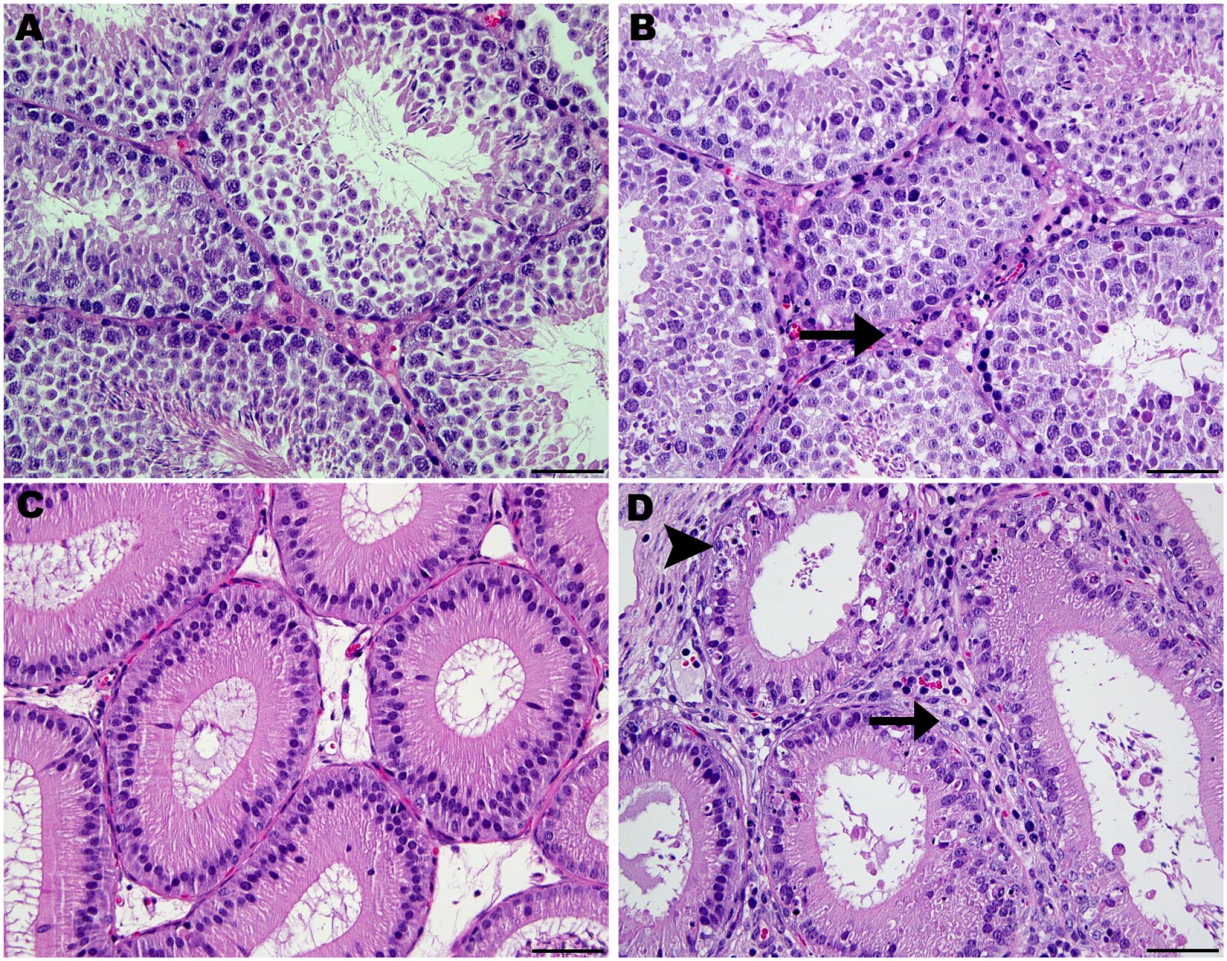
Normal histology and histopathology of the testicle and epididymis of control and Zika vius infected *Ifnar*^*−/−*^ males. (**A**) Normal testicle from a sham-infected male. The interstitium contain Leydig (interstitial) cells and blood vessels. (**B**) Testis from a Zika virus infected *Ifnar*^*−/−*^ male with mild orchitis. Mild neutrophilic infiltration (arrow) is observed in the interstitium. (**C**) Normal epididymis from a sham-infected male. (**D**) Epididymis from a Zika virus infected male exhibiting mild epididymitis. There is neutrophilic infiltration (arrow) of interstitium and individual epithelial cell necrosis (arrowhead). All sections 400x magnification; hematoxylin and eosin stain; bar = 50 µm.

Mild orchitis and epididymitis were the most severe inflammatory lesions observed in the testicle and epididymis of ZIKV-infected males (Fig. 1). No epididymal or testicular inflammation or epithelial cell necrosis was observed in sham-infected males. A focus of individual cell necrosis was observed in the seminal vesicle of a single ZIKV-infected male and mild and focal interstitial mononuclear inflammation was observed adjacent to the prostate of another sham-infected male. Statistical analysis was not performed on the histopathologic lesion severity score data as currently recommended by the International Harmonization of Toxicologic Pathology Nomenclature^30^. These data are provided in Supplemental Table 1.

**Figure 2:**
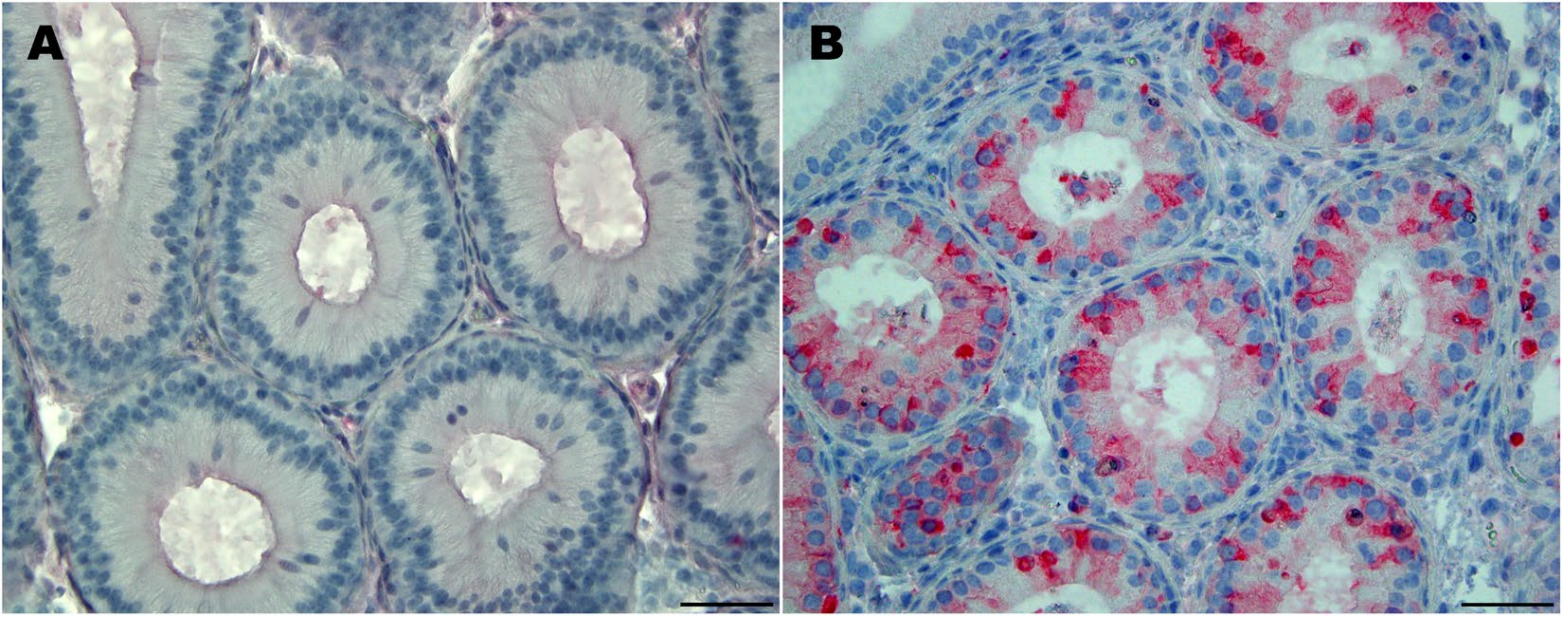
Immunohistochemical staining for Zika virus antigen in the epididymis. (**A**) Epididymis from a sham-infected male (400×). (**B**) Epididymis from a ZIKV-infected male. Focally, moderate numbers of epididymal epithelial cells exhibit cytoplasmic ZIKV antigen immunoreactivity (400×).

ZIKV antigen was detected by immunohistochemistry within epididymal epithelial cells of all ZIKV-infected males at 7 DPI (Fig. 2). Antigen was not detected in seminiferous tubules of the testicle, but low numbers of Leydig (interstitial) cells showed ZIKV antigen immunoreactivity.

### Quantitative RT-PCR Results

#### Artificial Insemination Fluid

**Figure 3:**
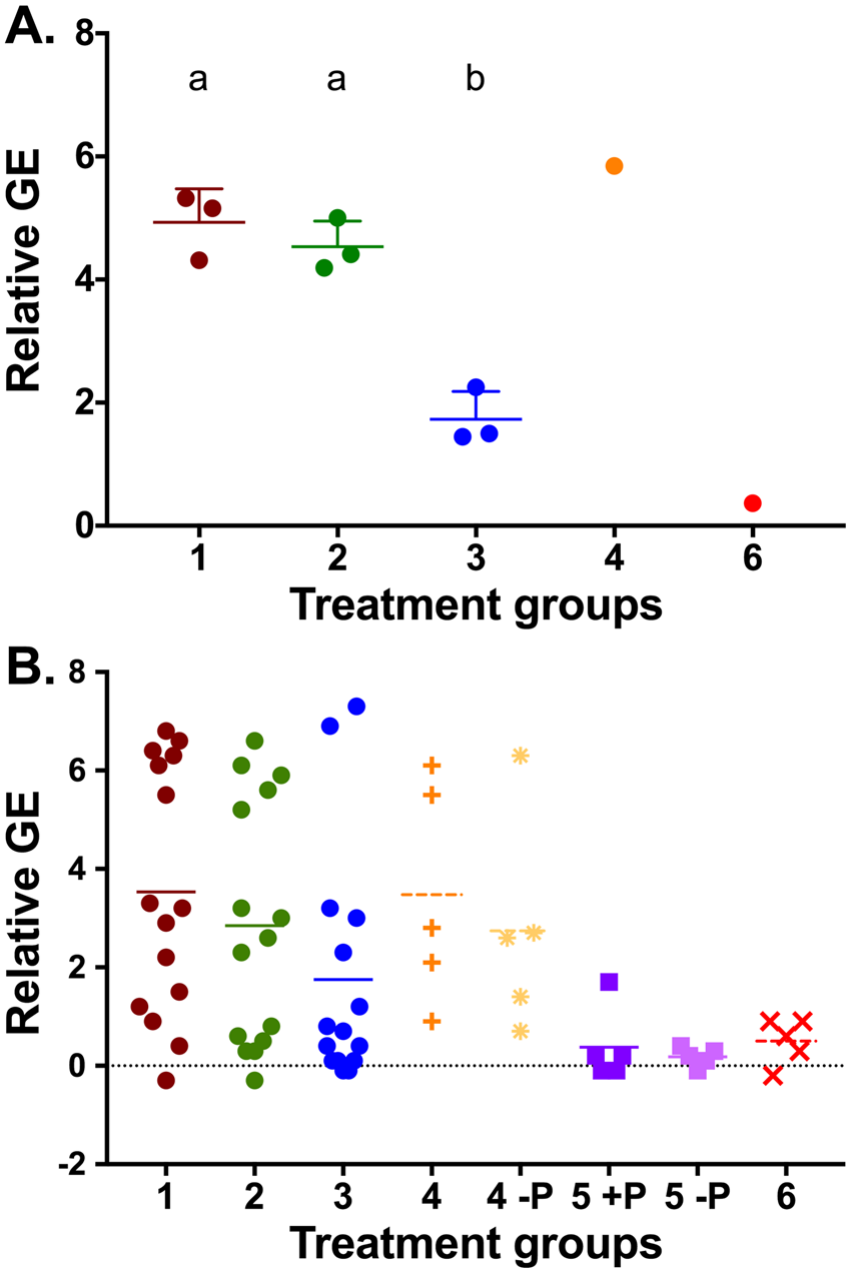
Relative Genome Equivalents of Zika Virus RNA (**A**) Relative genome equivalents of Zika virus (ZIKV) in the artificial insemination of treatment groups. Group 3 (b), epididymal flush from a sham-infected male and seminal plasma from a ZIKV-infected male, had statistically significant lower ZIKV RNA than groups 1 and 2 (a). (**B**) Relative genome equivalents of Zika virus in peripheral whole blood of intra-vaginally inoculated females. High viral RNA loads were observed in treatment groups one, two, three and four.+P = progesterone pre-treated; −P = no progesterone treatment.

ZIKV RNA was detected by qRT-PCR in every artificial insemination treatment except treatment 6 (Fig. 3). Relatively low concentrations of ZIKV RNA were detected in treatment 3, composed of EF- and SP+. Treatment 3 had a statistically significant (p = 0.02) lower viral RNA load compared to treatments 1 (EF+ and SP+) and 2 (EF+ and SP−). There was no significant difference (p = 0.18) between treatment groups 1 and 2. Treatment 4, EF− and SP− spiked with 10^4^ CCID_50_ ZIKV, resulted in detection of 5.8 relative genome equivalents of virus in the artificial insemination fluid.

#### Female Whole Blood

**Figure 4:**
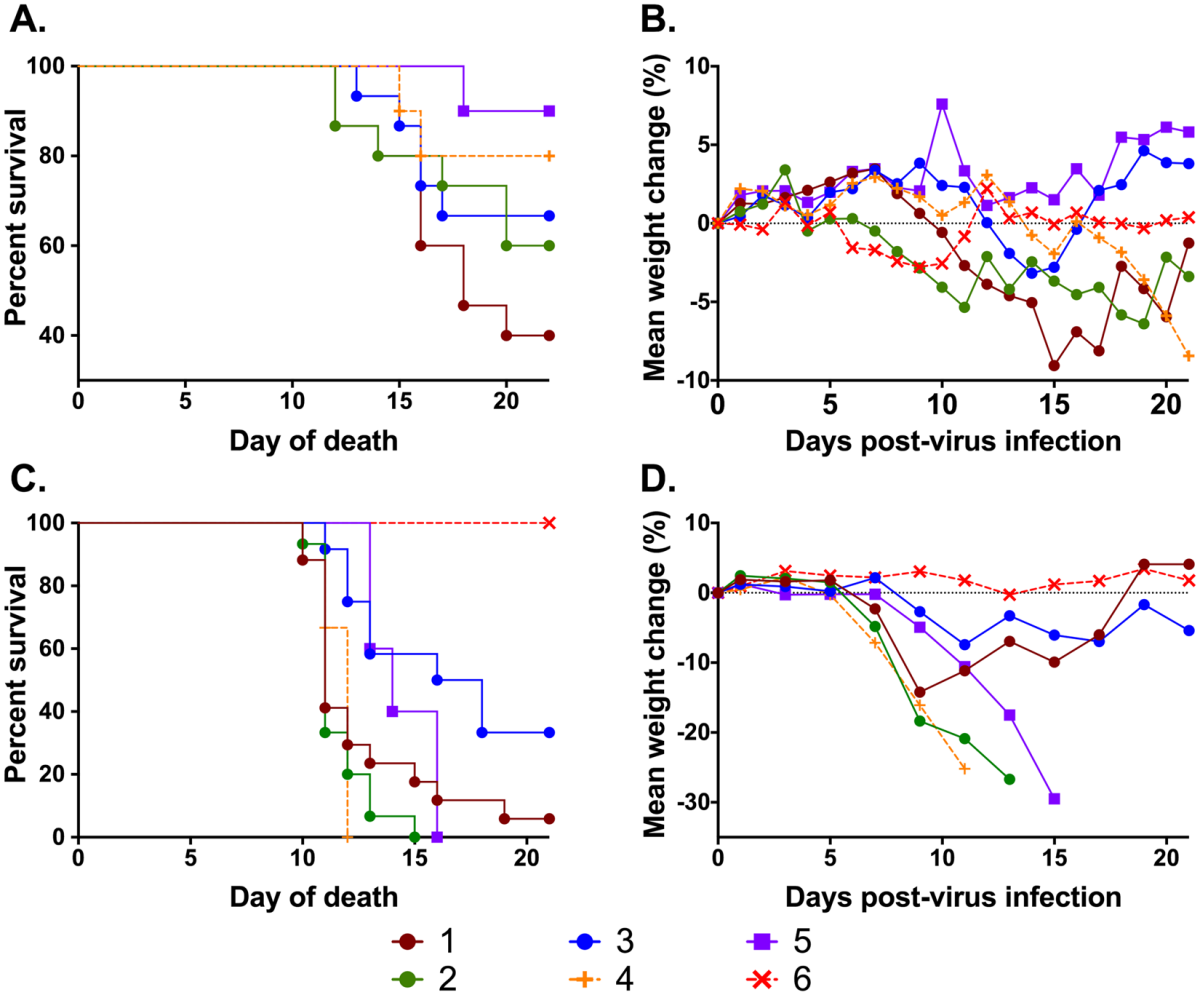
Female Morbidity and Mortality (**A**) Survival of intravaginally inoculated females in each treatment group. One hundred percent survival was observed in the negative control group, six. In addition, the non-progesterone treated replicates of treatments four and five had 100% survival. (**B**) Percent weight change of intravaginally inoculated females. Weight change was compared between the day of intravaginal inoculation and the weight on the day of death. An average weight loss was observed in treatments 1, 2, and 4. (**C**) Survival of subcutaneously inoculated females. Complete cohort survival was only observed in treatment 6, artificial insemination fluid derived from sham-infected males. One hundred percent mortality was observed in treatments 2, 4 and 5. (**D**) Percent weight change of subcutaneously inoculated females. Significant weight loss was observed in treatments 2, 4 and 5. Initial weight loss was observed in treatment 1 with surviving females recovering weight following days 11 post infection.

In order to show systemic spread of virus following intra-vaginal inoculation, peripheral whole blood samples were collected on all intra-vaginally infected females at 7 DPI (Table 2). ZIKV RNA was detected in at least one female from all replicates of treatment 1, 2 and 3 (Fig. 4). One replicate of treatment 3 (EF−, SP+) resulted in no detectable ZIKV RNA at 3 or 7 DPI (0%; n = 0/5). ZIKV RNA was detected in 20% (n = 1/5) progesterone treated females from treatment 5 and 0% (n = 0/5) of females of the same treatment that did not receive progesterone. ZIKV RNA was not detected at 7 DPI in the treatment 6 females (EF−, SP−). The presence of ZIKV RNA in whole blood samples at 7 days following subcutaneous injection was confirmed by qRT-PCR. Zika virus RNA was detected in all treatment groups except treatment 6 (Fig. 4).

**Table 2:** Percent of Females with Zika Virus Viremia at 7 Days Post Infection.

Treatment	Replicate 1	Replicate 2	Replicate 3	Average
**Intravaginal Inoculation Treatment**				
One (+EF/+SP)	100% (5/5)	80% (4/5)	60% (3/5)	80% (12/15)
Two (+EF/−SP)	40% (2/5)	40% (2/5)	100% (5/5)	60% (9/15)
Three (−EF/+SP)	100% (5/5)	20% (1/5)	0% (0/5)	40% (6/15)
Four (−EF/−SP; Spike)	80% (4/5); Progesterone	80% (4/5); Non-progesterone	NA	80% (8/10)
Five (MEM Spike)	20% (1/5); Progesterone	0% (0/5); Non-Progesterone	NA	10% (1/10)
Six (−EF/−SP)	0% (0/5)	NA	NA	0% (0/0)
**Subcutaneous Inoculation Treatment**				
One (+EF/+SP)	100% (3/3)	66.66% (2/3)	100% (3/3)	88.88% (8/9)
Two (+EF/−SP)	100% (3/3)	100% (3/3)	100% (3/3)	100% (9/9)
Three (−EF/+SP)	100% (3/3)	66.66% (2/3)	66.66% (2/3)	77.77% (7/9)
Four (−EF/−SP; Spike)	100% (3/3); Progesterone	NA	NA	100% (3/3)
Five (MEM Spike)	100% (3/3); Progesterone	100% (2/2); Non-Progesterone	NA	100% (5/5)
Six (−EF/−SP)	0% (0/3)	NA	NA	0% (0/3)

Average percent of females with ZIKV RNA detected in peripheral whole blood at 7 days post intra-vaginal and subcutaneous inoculation. EF = epididymal flush; SP = seminal plasma; + = Zika virus infected; − = Sham; MEM = Minimal Essential Media; NA = not applicable.

### Estrous Cycle and Effect of Hormone Therapy on Survival

Estrous stage was determined via vaginal cytology by a veterinary pathologist (CSC) prior to inoculation. The results are summarized in Table 3.

**Table 3:** Estrous Cycle of Females at the Time of Zika Virus Intravaginal Inoculation

Treatment	Pro-Estrus	Estrus	Metestrus	Diestrus
One (+EF/+SP)	33.3% (5/15)	6.7% (1/15)	6.7% (1/15)	40.0% (6/15)
Two (+EF/Sham SP)	26.7% (4/15)	6.7% (1/15)	0% (0/15)	66.7% (10/15)
Three (Sham EF/+SP)	26.7% (4/15)	6.7% (1/15)	6.7% (1/15)	60.0% (9/15)
Four (Sham EF/Sham SP; Spiked with 5 × 10^4^ ZIKV)	30.0% (3/10)	40.0% (4/10)	10.0% (1/10)	20.0% (2/10)
Five (MEM* Solution 5 × 10^4^ ZIKV) Progesterone Treated	0% (0/5)	0% (0/5)	40.0% (2/5)	60.0% (3/5)
Five (MEM* Solution 5 × 10^4^ ZIKV) No Progesterone	0% (0/5)	40.0% (2/5)	0% (0/5)	60.0% (3/5)
Six (Sham EF/Sham SP)	60.0% (3/5)	0% (0/5)	20.0% (1/5)	20.0% (1/5)

EF = epididymal flush; SP = seminal plasma; + = Zika virus infected; − = Sham; MEM = Minimal Essential Media.

### Female Morbidity and Mortality

Morbidity was determined as a percentage of weight-loss relative to the initial weight on the day of intravaginal inoculation. Four groups of females showed weight gain over the course of the study including the treatment 6 (EF−, SP−) control. The three remaining cohorts that showed positive weight gain included two groups within treatment 3 (EF−, SP+). The final replicate that showed weight gain was the non-progesterone treated replicate of treatment 5 (MEM-spiked).

Female AG129 mice were followed for 21 DPI for mortality. Humane euthanasia was performed on animals exhibiting 20% body weight loss or severe neurologic abnormalities including bilateral, hind limb paresis and paralysis or continuous circling. One hundred percent mortality was observed in one replicate of treatments 1 (EF+, SP+) and 3 (EF−, SP+).

Only one female (20%) receiving subcutaneous progesterone and (treatment 5), 10^4^ CCID_50_ of ZIKV in MEM solution intra-vaginally without the addition of EF or SP died during the course of the study (17 DPI). No females receiving 10^4^ CCID_50_ of ZIKV in MEM solution intra-vaginally, without the addition of EF, SP or subcutaneous progesterone, died.

In the subcutaneous inoculation model, weight-loss was observed in every treatment group relative to treatment 6 (Fig. 4). Complete cohort survival was only observed in treatment 6, and all mice succumbed to infection by 21 in treatments 2, 4 and 5 (Fig. 4).

## Discussion

Zika virus is an emerging arbovirus for which increasing evidence suggests the potential for sexual transmission of infection. The tissue of origin of infectious virions within the male reproductive tract has not been elucidated. Here, we demonstrate ZIKV RNA and antigen in male reproductive tissue and show that infectious virions can be derived from either epididymal or accessory sex gland epithelial cells and can be transmitted via intravaginal inoculation. Additionally, we show that pre-treatment of naïve females with progesterone is critical in establishing intra-vaginal ZIKV infection in females.

As previously shown, we corroborate that ZIKV antigen or RNA can be detected within all components of the male reproductive tract of mice^17^. ZIKV RNA was detected by qRT-PCR in the artificial insemination fluid from every experimentally inoculated group regardless of which segment of the reproductive tract was utilized to create the insemination fluid (EF or SP). Both EF and SP derived from ZIKV-infected males was capable of causing ZIKV viremia in naïve AG129 females when females were inoculated intravaginally or subcutaneously, indicating that infectious ZIKV was present in both EF and SP.

One critical difference between the current study and previous intravaginal inoculation studies is the use of male-derived reproductive fluid rather than cell-free virus derived from cell culture. Macaques have been shown to be susceptible to infection after intravaginal inoculation of cell-free, culture derived ZIKV^31,32^. While the macaque has been shown to be susceptible, previous studies have used viral challenge doses around 10^7^ PFU and at least one model used continuous, weekly, intravaginal inoculation until ZIKV was detected by PCR^32^. The present study uses a single inoculation of 10^4^ PFU as a positive control for virus exposure at a known virus challenge dose. This is the first study to highlight that infectious virions are produced in the male reproductive tract and are capable of being sexually transmitted to females resulting in peripheral viremia.

ZIKV antigen was detected in sections of epididymis but not seminal vesicle or prostate. This may be due to a lower viral load in accessory sex gland tissue resulting in viral antigen being produced below the limit of detection. Artificial insemination fluid in which only the SP was derived from a ZIKV-infected male (treatment 3) had statistically significant (p = 0.02) lower ZIKV RNA loads than groups in which the EF was derived from ZIKV-infected males (treatments 1 and 2). A single replicate of treatment 3 had 100% mortality in intravaginally inoculated females. Mortality was observed in all replicates of treatment 3 when the artificial insemination fluid was injected subcutaneously (Fig. 4). This data suggests that higher viral loads are required to create systemic disease with an intravaginal inoculation route compared to subcutaneous inoculation. This data also suggests that while SP can serve as a source of infectious virus in the ejaculate, there is significant biologic variability in the amount of infectious virus contained within the SP at 7 DPI in *Ifnar*^*−/−*^ mice. In the current study, there was variability in the rate of detection of peripheral viremia in intravaginally inoculated females. This variability is attributed to unidentified, uncontrolled biologic variability in the model and has been previously reported in macaque models of intravaginal ZIKV inoculation^32^. The method of SP preparation could have partially contributed to this biologic difference. These results suggest that screening the ejaculate from sperm donors or testing for concerned patients attempting a pregnancy following trips to endemic regions may be difficult to interpret as presence of viral RNA did not guarantee systemic infection in the recipient female.

The presence of SP was associated with increased disease and presence of viremia in intra-vaginally inoculated AG129 females (Fig. 4). Seminal plasma is hypothesized to downregulate the immune response at the time of insemination^10^. Immunosuppression in the face of reproductive tract infection has significant clinical consequences, both in pregnant and non-pregnant women. This data suggests that SP plays a crucial role in immunosuppression of the vaginal mucosa allowing both localized and systemic ZIKV infection.

In mouse models of ZIKV infection of the male reproductive tract, ZIKV antigen is co-localized with an inflammatory response^17,33^. Yet in humans, clinical orchitis, epididymitis and prostatitis are not reported to occur. In the current study, histopathology severity scores revealed minimal to mild epididymitis in ZIKV-infected males. Individual cell necrosis was observed in low numbers of tubules of males receiving a mild score, but epithelial cell necrosis was not observed in males receiving a minimal score. Viremia was observed in females inoculated with EF from males receiving either minimal or mild scores, suggesting that cellular necrosis does not need to occur in order to release infectious virions into the ejaculate. Instead, release of infections virions may be done through epididymosome release resulting in minimal cellular damage or initiation of inflammation.

Progesterone pre-treatment increased mortality in both SP and non-SP treatment groups (Supplemental Tables 2 and 3). Progesterone has been shown to decrease lymphocyte function^19^, and was used to temporarily mimic T-lymphocyte suppression associated with pregnancy^34^. No mortality was observed in the non-progesterone treated females intravaginally challenged with 10^4^ CCID_50_ of ZIKV in MEM (treatment 4 and 5), regardless of estrous cycle stage (Supplemental Table 2). This is in contrast to a report in which diestrus was associated with increased morbidity and mortality relative to estrus^24^. In the study by Tang *et al*., a supra-physiologic dose of progesterone was utilized to induce diestrus in intra-vaginally inoculated females, and the conclusions were attributed to estrous cycle rather than exposure to progesterone. We believe the difference in the results is due to exposure to a supra-physiologic dose of progesterone. Viremia, morbidity and mortality were observed regardless of the stage of estrous at the time of intravaginal inoculation. Limited morbidity and survival until 21 DPI was observed in all estrous phases in progesterone treated animals. Non-progesterone treated females in diestrus under natural conditions did not exhibit viremia, morbidity or mortality throughout the study (Supplemental Table 3). These results suggest that exposure to supra-physiologic doses of progesterone may down-regulate the uterine and vaginal mucosal immune response and pre-dispose to sexual transmission of viral infections. Lymphocytes from gravid animals are more sensitive to progesterone than non-gravid animals^19^, and pregnancy in humans may also pre-dispose to sexual transmission of ZIKV.

We have previously shown that epithelial cells from the epididymis and accessory sex glands are susceptible to ZIKV infection^17^. Here we show that infectious ZIKV can be produced in either EF or SP, confirming that both tissues support ZIKV replication. In addition, presence of infectious ZIKV in EF and SP suggests a potential route of ZIKV movement from the bloodstream into the male reproductive tract that circumnavigates the blood-testicle barrier. We hypothesized that infectious virions could be produced in the epididymis allowing retrograde movement of ZIKV from the epididymis to the testicle. Here we show that the contents of the epididymis at 7 DPI are infectious and may serve as a source for further infection in the male reproductive tract. Contrary to the epididymis, limited pathology is observed in the accessory sex glands, specifically the prostate and seminal vesicles. ZIKV infection results in low level production of infectious virions and mild pathology in the accessory sex glands. We hypothesize that prostasomes may be utilized by ZIKV for release in the ejaculate without initiating a significant inflammatory response.

Further work to identify the source of viral persistence in the male reproductive tract and to clarify the presence of infectious viral particles in the chronic phase of disease will assist in the development of anti-viral therapies and better target diagnostic screening modalities to prevent sexual transmission and congenital abnormalities.

## Electronic supplementary material

Supplementary tables
