# Supplementary material for: Coitus-Free Sexual Transmission of Zika Virus in a Mouse Model
Source: Sci Rep. 2018 Oct 18;8:15379. doi: 10.1038/s41598-018-33528-2 (PMC6194026; doi:10.1038/s41598-018-33528-2)
Supplement: Supplementary file 1 — Supplementary tables [file 41598_2018_33528_MOESM1_ESM.pdf]

Supplementary Data

Coitus-Free Sexual Transmission of Zika Virus in a Mouse Model

Chad S. Clancy[1,2], Arnaud J. Van Wettere[1], John D. Morrey[2], Justin G. Julander[2]

[1]Utah Veterinary Diagnostic Laboratory, School of Veterinary Medicine, Department of Animal, Dairy, and Veterinary Sciences, Utah State University, Logan, Utah, United States of America

[2]Institute for Antiviral Research, Department of Animal, Dairy, and Veterinary Sciences, Utah State University, Logan, Utah, 84322-5600, United States of America

**Supplemental Table 1**. Histopathology Severity Score of Male Reproductive Tissue										
**Treatment**	**Negative**		**Minimal**		**Mild**		**Moderate**		**Severe**	
	Epididymis	Testicle	Epididymis	Testicle	Epididymis	Testicle	Epididymis	Testicle	Epididymis	Testicle
One (EF+/SP+)	0% (0/3)	33% (1/3)	33% (1/3)	33% (1/3)	66% (2/3)	33% (1/3)	0% (0/3)	0% (0/3)	0% (0/3))	0% (0/3)
Two (EF+/SP-)	0% (0/3)	66% (2/3)	66% (2/3)	33% (1/3)	33% (1/3)	0% (0/3)	0% (0/3)	0% (0/3)	0% (0/3)	0% (0/3)
Three (EF-/SP+)	100% (3/3)	100% (3/3)	0% (0/3)	0% (0/3)	0% (0/3)	0% (0/3)	0% (0/3)	0% (0/3)	0% (0/3)	0% (0/3)
Four (EF-/SP-; Spike)	100% (2/2)	100% (2/2)	0% (0/2)	0% (0/2)	0% (0/2)	0% (0/2)	0% (0/2)	0% (0/2)	0% (0/2)	0% (0/2)
Six (EF-/SP-)	100% (1/1)	100% (1/1)	0% (0/1)	0% (0/1)	0% (0/1)	0% (0/1)	0% (0/1)	0% (0/1)	0% (0/1)	0% (0/1)

Supplemental Table 1. The lesion severity of the testicle and epididymis. Lesions in both the testicle and epididymis at 7 days post infection were minimal to mild in all infected males. Inflammation was not observed in sham-infected males. EF= epididymal flush; SP= seminal plasma; += Zika virus infected; -= Sham.

**Supplemental Table 2.** Effect of Estrous Cycle on Female Early Euthanasia				
**Hormone Treatment**	**Estrous Cycle**			
	**Proestrus**	**Estrus**	**Metestrus**	**Diestrus**
**Progesterone**	39.1% (9/23)	8.7% (2/23)	8.7% (2/23)	43.5% (10/23)
**Sham Treatment**	0% (0/0)	0% (0/0)	0% (0/0)	0% (0/0)

**Supplemental Table 3.** Effect of Estrous Cycle on Female Survival				
**Hormone Treatment**	**Estrous Cycle**			
	**Proestrus**	**Estrus**	**Metestrus**	**Diestrus**
**Progesterone**	26.7% (8/30)	6.7% (2/30)	6.7% (2/30)	60% (18/30)
**Sham Treatment**	0% (0/10)	50% (5/10)	10% (1/10)	40% (4/10)
